# Supplementary material for: Behavioural Responses of Defended and Undefended Prey to Their Predator—A Case Study of Rotifera
Source: Biology (Basel). 2022 Aug 13;11(8):1217. doi: 10.3390/biology11081217 (PMC9404713; doi:10.3390/biology11081217)
Supplement: Supplementary file 1 [file biology-11-01217-s001.zip › biology-1799513-supplementary.pdf]

SUPPLEMENTARY MATERIALS

Behavioural responses of defended and undefended prey to their predator- a case study of rotifera

Victor Parry, Ulrike E. Schlägel, Ralph Tiedemann & Guntram Weithoff

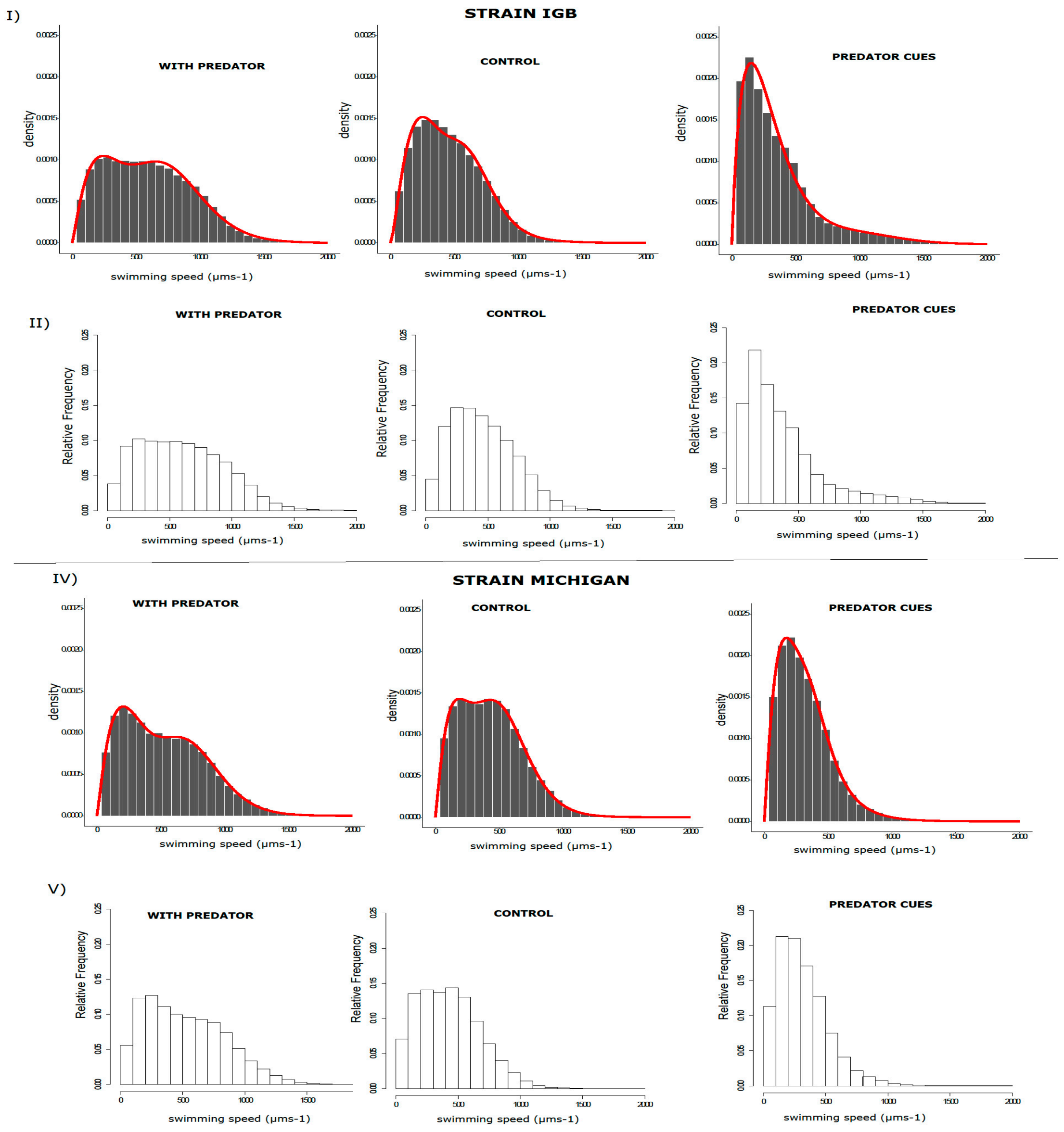

Figure S1: Plot showing density and relative frequency plots of swimming speed (µm s^{-1}) of unspined *B. calyciflorus* strains in different treatments. I) density plot of strain "IGB" II) relative frequency plot of strain "IGB" III) density plot of strain "Michigan" IV) relative frequency plot of strain "Michigan"

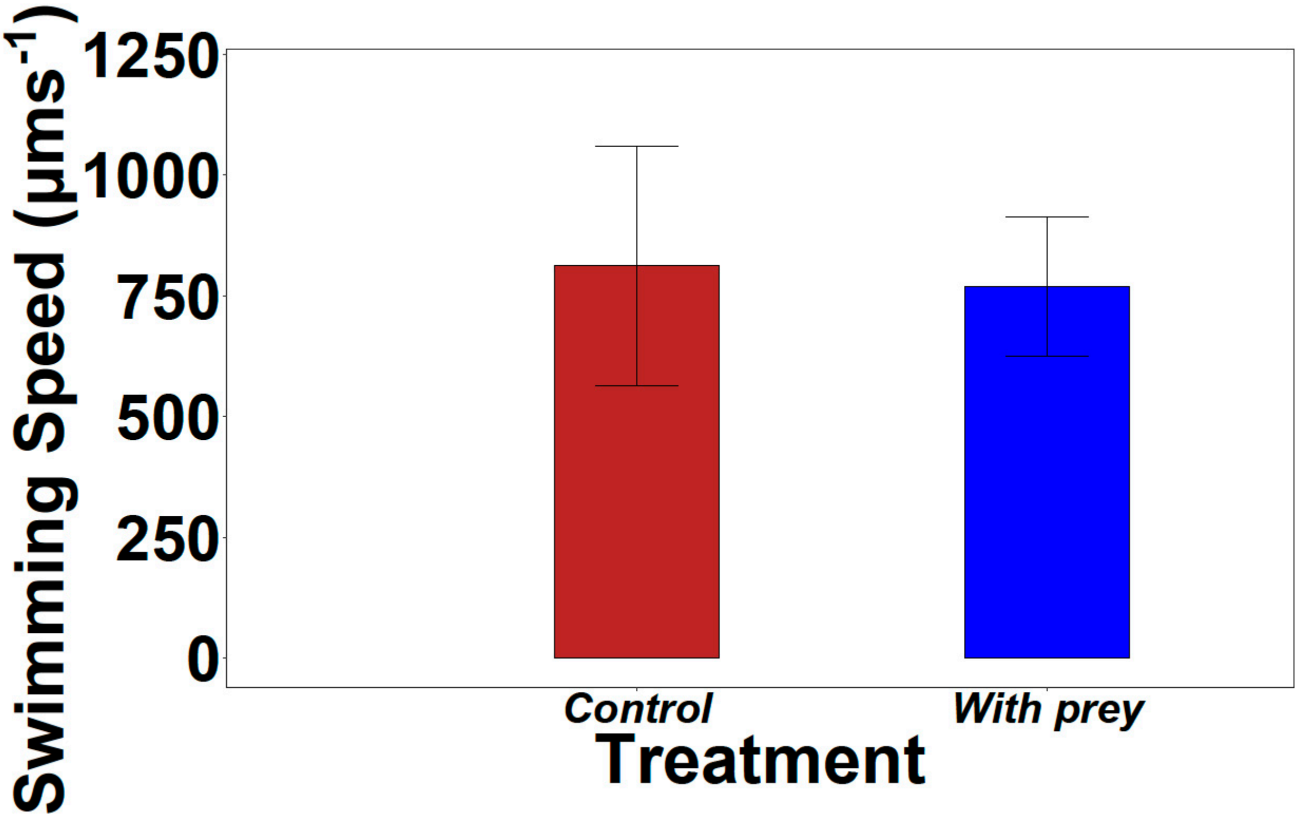

Figure S2: I) Swimming speed (μm s^{-1}) ± SD of *A. brightwellii* in different treatments with spined *Brachionus calyciflorus*. Control represents control experiment with *A. brightwellii* alone in the medium. With prey represents the experiment with *B. calyciflorus* and *A. brightwellii* in the same

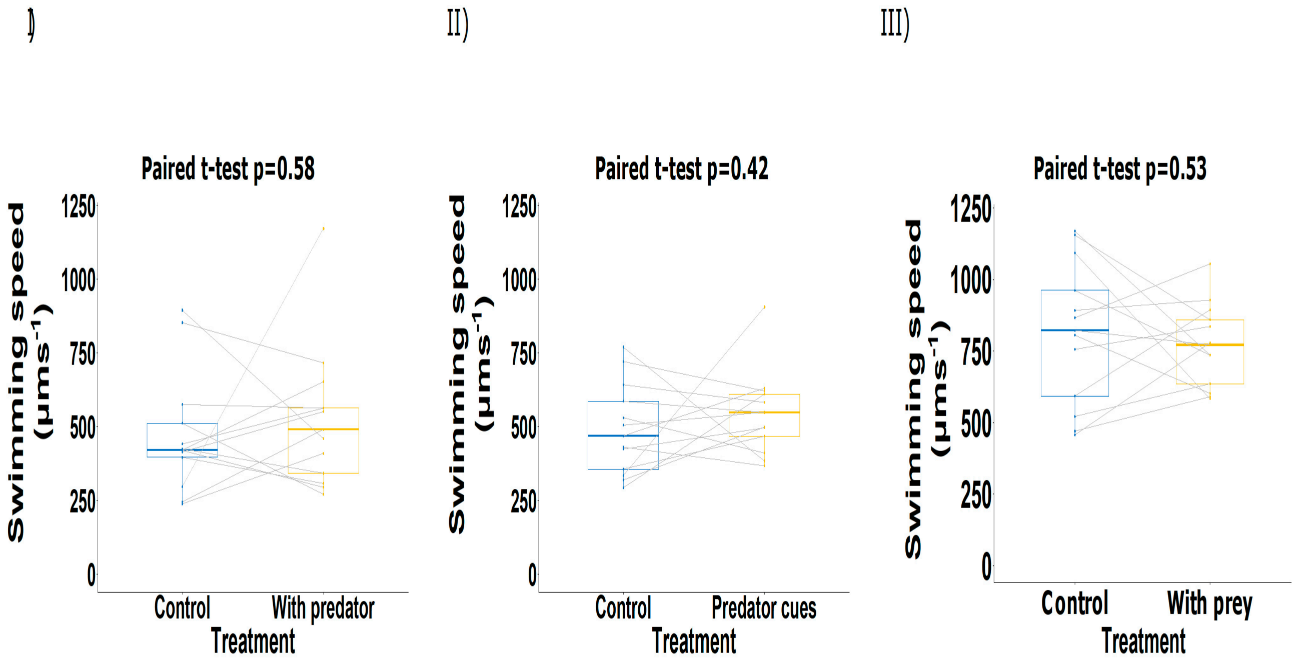

Figure S3: Plot showing t-test between repeated treatments of *spined Brachionus calyciflorus* and predator A. *brightwellii* swimming speed I) Control and With predator treatment of spined *Brachionus calyciflorus* II) Control and Predator cues (**Kairomones**) spined *Brachionus calyciflorus* III) Control and with spined prey of *A. brightwellii*

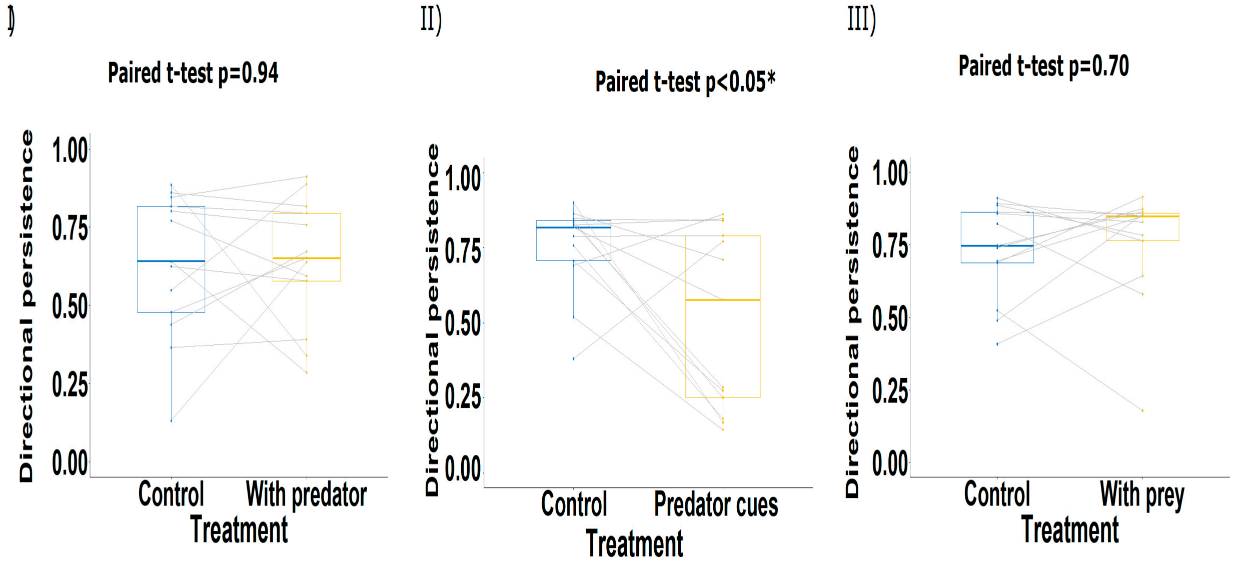

Figure S4: Plot showing t-test between repeated treatments of spined *Brachionus calyciflorus* and predator *A. brightwellii* directional persistence I) Control and With predator treatment of spined *Brachionus calyciflorus* II) Control and Predator cues (**Kairomones**) spined *Brachionus calyciflorus* III) Control and with spined prey of *A. brightwellii.*

Table S1. MANCOVA analyses of unspined *B. calyciflorus* strain “IGB” with treatment as factor and survival (number of prey) as covariate and swimming speed (µms-^{1}) and directional persistence as dependent variables, P < 0.001 (***), P < 0.01 (**) and P value <0.05* indicates significance.

Multivariate Tests

	**Test**	**value**	**F**	**df1**	**df2**	**p**
**treatment**	Pillai's Trace	0.563	8.04	4	82	<0.001***
**survival**	Pillai's Trace	0.047	0.047	2	40	0.377

Univariate Tests

	**Dependent varable**	**Sum of squares**	**F**	**df**	**Mean square**	**p**
**treatment**	Swimming speed	305354.536	11.064	2	152677.268	<0.001***
	Directional persistence	0.405	4.752	2	0.202	0.0139*
**survival**	Swimming speed		0.0015	1	21.561	0.969
	Directional persistence	565732.49	1.686	1	0.072	0.201
**Residuals**	Swimming speed	0.072		41	13798.35	
	Directional persistence	1.7451		41	0.042	

Table S2. MANCOVA analyses of unspined *B. calyciflorus* strain "Michigan" with treatment as factor and survival (number of prey) as covariate and swimming speed (µms-[1]) and directional persistence as dependent variables, P < 0.001 (***), P < 0.01 (**) and P value <0.05* indicates significance

Multivariate Tests

	**Test**	**value**	**F**	**df1**	**df2**	**p**
**treatment**	Pillai's Trace	0.571	8.186	4	82	<0.001***
**survival**	Pillai's Trace	0.057	1.198	2	40	0.312

Univariate Tests

	**Dependent varable**	**Sum of squares**	**F**	**df**	**Mean square**	**p**
**treatment**	Swimming speed	315558.648	15.565	2	157779.324	<0.001***
	Directional persistence	0.1842	3.176	2	0.092	0.052
**survival**	Swimming speed	1043.141	0.102	1	1043.141	0.749
	Directional persistence	0.0529	1.825	1	0.0529	0.184
**Residuals**	Swimming speed	415606.686		41	10136.748	
	Directional persistence	1.189		41	0.029	

Table S3. Regression analysis of mean swimming speed (μm s^{-1}), relative swimming speed (BLs^{-1}) and directional persistence of spined *B. calyciflorus* "Michigan" with different spine lengths and body lengths in laboratory experiments, P < 0.001 (***), P < 0.01 (**) and P value <0.05* indicates significance

Treatment	Sub-treatment	Variable	df	N	F	intercept±SE	slope±SE	r^2	p-value
**Live Predator**	Control	Spine length(μm) vs Body length (μm)	11	13	6.81	173±30	0.6±0.2	0.38	0.024*
		Swimming speed (μms-^{1}) vs Body length (μm)	11	13	6.94	1381±330	-3±1	0.39	0.023*
		Swimming speed (μms-^{1}) vs Spine length (μm)	11	13	0.11	591±216	-0.5±2	0.01	0.745
		Relative swimming speed (BLs-^{1}) vs Body length (μm)	11	13	14.02	9±2	-0.02±0.01	0.56	0.003**
		Relative swimming speed (BLs-^{1}) vs	11	13	0.74	3±16	-008±0.01	0.54	0.408

		Spine length (µm)							
		Persistence vs Body length (µm)	11	13	2.68	1.21±0.36	-0.02±0.001	0.20	0.130
		Persistence vs Spine length (µm)	11	13	0.13	0.56 ±0.21	0.005±0.001	0.01	0.730
**Live Predator**	With predator	Spine length(µm) vs Body length (µm)	11	13	6.81	173±30	0.6±0.2	0.38	0.024*
		Swimming speed (µms^{-1}) vs Body length (µm)	11	13	5.64	1148±289	-3±1	0.34	0.037*
		Swimming speed (µms^{-1}) vs Spine length (µm)	11	13	0.91	631±175	-1±1	0.08	0.361
		Relative swimming speed (µms^{-1}) vs Body length (µm)	11	13	2.694	8±2	-0.03±0.01	0.55	0.004**

		Relative swimming speed (μms^{-1}) vs Spine length (μm)	11	13	2.0	4±1	-0.01±0.01	0.16	0.181
		Persistence vs Body length (μm)	11	13	4.25	1.22±0.31	-0.002±0.001	0.25	0.080
		Persistence vs Spine length (μm)	11	13	5e-5	$6x10^{-1}±1x10^{-1}$	$-2 x10^{-5}±1x10^{-3}$	2e-03	0.880
**Predator cues**	Control	Spine length(μm) vs Body length (μm)	11	13	5.4	88±27	0.7±0.4	0.19	0.136
		Swimming speed (μms^{-1}) vs Body length (μm)	11	13	5.4	1161±268	-4±2	0.33	0.041*
		Swimming speed (μms^{-1}) vs Spine length (μm)	11	13	0.15	852±226	-5±4	0.01	0.196
		Relative swimming	11	13	17.2	14±3	0.07±0.02	0.61	0.002**

		speed (BLs^{-1}) vs Body length (µm)							
		Relative swimming speed (BLs^{-1}) vs Spine length (µm)	11	13	2.97	8±2	-0.06±0.04	0.21	0.113
		Persistence vs Body length (µm)	11	13	3.08	1.29±0.31	-0.004 ± 0.002	0.22	0.107
		Persistence vs Spine length (µm)	11	13	3.05	1.14±0.23	-0.006±0.004	0.22	0.108
**Predator cues**	Predator cues	Spine length(µm) vs Body length (µm)	11	13	5.4	88±27	0.7±0.4	0.19	0.136
		Swimming speed (μms^{-1}) vs Body length (µm)	11	13	2.7	-39±322	4±2	0.20	0.125
		Swimming speed (μms^{-1}) vs	11	13	7e-4	498±270	-0.1±4	$7x10-^{5}$	0.979

	Spine length (µm)							
	Relative swimming speed (BLs^{-1}) vs Body length (µm)	11	13	7e-4	4±3	0.002±0.02	7x10-^{4}	0.932
	Relative swimming speed (BLs^{-1}) vs Spine length (µm)	11	13	0.21	5±2	-0.01±0.03	0.019	0.650
	Persistence vs Body length (µm)	11	13	2.26	-0.43 ± 0.64	0.007 ± 0.005	0.17	0.161
	Persistence vs Spine length (µm)	11	13	4.31	-0.40 ± 0.44	0.014 ± 0.007	0.28	0.062
